# The physiological basis of neurorehabilitation - locomotor training after spinal cord injury

**DOI:** 10.1186/1743-0003-10-5

**Published:** 2013-01-21

**Authors:** Michèle Hubli, Volker Dietz

**Affiliations:** 1 Spinal Cord Injury Center, Balgrist University Hospital, University of Zurich, Forchstrasse 340, 8008, Zurich, Switzerland

**Keywords:** Locomotion, Neurorehabilitation, Neuronal plasticity, Spinal cord injury

## Abstract

Advances in our understanding of the physiological basis of locomotion enable us to optimize the neurorehabilitation of patients with lesions to the central nervous system, such as stroke or spinal cord injury (SCI). It is generally accepted, based on work in animal models, that spinal neuronal machinery can produce a stepping-like output. In both incomplete and complete SCI subjects spinal locomotor circuitries can be activated by functional training which provides appropriate afferent feedback. In motor complete SCI subjects, however, motor functions caudal to the spinal cord lesion are no longer used resulting in neuronal dysfunction. In contrast, in subjects with an incomplete SCI such training paradigms can lead to improved locomotor ability. Appropriate functional training involves the facilitation and assistance of stepping-like movements with the subjects’ legs and body weight support as far as is required. In severely affected subjects standardized assisted locomotor training is provided by body weight supported treadmill training with leg movements either manually assisted or moved by a driven gait orthosis. Load- and hip-joint related afferent input is of crucial importance during locomotor training as it leads to appropriate leg muscle activation and thus increases the efficacy of the rehabilitative training. Successful recovery of locomotion after SCI relies on the ability of spinal locomotor circuitries to utilize specific multisensory information to generate a locomotor pattern. It seems that a critical combination of sensory cues is required to generate and improve locomotor patterns after SCI. In addition to functional locomotor training there are numbers of other promising experimental approaches, such as tonic epidural electrical or magnetic stimulation of the spinal cord, which both promote locomotor permissive states that lead to a coordinated locomotor output. Therefore, a combination of functional training and activation of spinal locomotor circuitries, for example by epidural/flexor reflex electrical stimulation or drug application (e.g. noradrenergic agonists), might constitute an effective strategy to promote neuroplasticity after SCI in the future.

## Introduction

A spinal cord injury (SCI) is a devastating event that, depending on the level and severity, impacts sensorimotor and autonomous function. In affected subjects the goal of rehabilitative interventions is the regaining of independence and thus a good quality of life. From the patients perspective this is probably best achieved by targeting restoration of bladder and bowel function, and in tetraplegic subjects upper limb function [1]. However, recovery of locomotor ability is also of high priority by SCI subjects independently from the severity, time after injury and age at the time of injury [2]. It is now widely accepted that the central nervous system is able to recover locomotor function following incomplete SCI with functional training on a treadmill combined with partial body weight support [3-5]. However, the physiological requirements for training effects remain vague. A century of research into the organization of the neuronal processes underlying the control of locomotion in mammals has demonstrated that the basic neuronal circuitries responsible for generating efficient stepping patterns are embedded within the lumbosacral spinal cord [6,7]. These spinal locomotor circuitries appear to play a crucial role in stepping ability in animal models and in human SCI. This review covers the physiological basis of effective locomotor training after SCI. Several studies in animal models, especially in rats and cats, have unraveled the neuronal principles underlying neurorehabilitation of locomotion after SCI. The role of neuronal plasticity will be discussed in this review. After SCI neuroplasticity in the cortex, brainstem and spinal cord can be exploited by rehabilitative approaches. This review will mainly focus on studies of neuroplastic changes at the spinal level as the knowledge gained from these studies is used to create novel translatory neurorehabilitative approaches with the aim to restore and improve locomotor ability after human SCI.

### Neuronal basis of human locomotion

The question, how does the central nervous system coordinate limb movements during locomotion in a seemingly “simple” and automatic manner challenges neuroscientists for more than a century. At the beginning of the last century (1911) Graham-Brown postulated his “half-center” hypothesis which demonstrated the intrinsic capacity of the mammalian spinal cord to generate rhythmic motor patterns without descending or sensory input [8]. Subsequently Grillner called these spinal neuronal circuitries central pattern generators (CPGs). CPGs are embedded within the lumbosacral spinal segments and are capable of generating stepping-like activation patterns [6]. However, a CPG alone does not appear to be sufficient for over-ground walking. Locomotion represents the interaction between the innate pattern and an appropriate modulation of leg muscle activation which has to continuously adapt to the present requirements, e.g., to the over-ground conditions. Feedback from a variety of sources, e.g., visual, vestibular and proprioceptive systems, is interpreted by and then integrated into the activity of the CPG [9]. The CPG can open and close reflex pathways in a context- and task-dependent manner. The sensory feedback and the context-specific requirements of the motor task determine the mode of organization of muscle synergies [10]. Additionally, supraspinal control is needed to provide both the drive for locomotion as well as the coordination to interact with a complex environment. Corticospinal access to locomotion control in humans is phase-dependent [11]. Brain centers can initiate CPG activity but the fundamental rhythmicity is hard-wired. For example, in the cat, it was shown that application of clonidine, a substance mimicking the action of long descending pathways, results in distinct and consistent alternating bursts of electromyographic (EMG) activity which induce spinal stepping [12]. In humans, neuroimaging methods have revealed that distinct cortical areas, e.g., both the medial primary sensory-motor cortices and the supplementary motor areas, become activated during locomotion [13,14] and the size of the activated areas is related to the subjects’ walking speed [15]. In addition, more demanding tasks, such as walking over obstacles, require more cortical control, especially during swing phase of stepping in humans [16] and the cat [17].

Quadrupedal locomotion is characterized by the coordination of forelimb and hindlimb rhythmic activities generated by common spinal neuronal control mechanisms, i.e., long propriospinal neurons coupling the cervical and lumbar segments [18,19]. This neuronal coupling and coordination of upper and lower limbs that is present in quadrupedal locomotion is preserved in bipedal gait. Unilateral tibial nerve stimulation during locomotion, but not during sitting or standing, leads to reflex responses in leg muscles and in the proximal muscles of both arms [20,21]. Such task-dependent coupling of thoraco-lumbar and cervical locomotor centers is flexible and allows humans to use the upper limb for fine, skilled movements, or alternatively for locomotor tasks, such as swimming or crawling, or for the control of body equilibrium during stepping, e.g., arm swing as a residual function of quadrupedal locomotion [22].

It is important that the neuronal mechanisms underlying human locomotor control in the normal and pathophysiological condition are understood, as it is only then that it is possible to maximize the recovery of locomotion in patients following central nervous system damage.

### Focusing on neuronal plasticity brought about by locomotor training

Modern neurorehabilitation no longer aims to simply compensate for disabilities in SCI subjects, rather it aims to functionally regain locomotor ability by exploiting neural plasticity and/or neural repair. A first question to ask is, “What is neuronal plasticity?” An example of neuronal plasticity is the spontaneous reorganization that is observed after SCI at the cortical level which can occur over one year [23]. Next to spontaneously occurring neuronal plasticity, it can also be induced by locomotor training at cortical level [24]. Plastic changes in sensorimotor cortex activity are related to functional locomotor recovery after an SCI [25]. Besides cortical plasticity it seems that other supraspinal centers, such as the cerebellum and brainstem are also important sites of neuronal plasticity in humans receiving locomotor training after SCI [24]. In humans, it is assumed that supraspinal plasticity is associated with plasticity of spinal neuronal circuits, but the evidence for this is predominantly from animal models of SCI [26].

Spinal neuronal circuits below the level of lesion can be activated by an appropriate afferent input, and this is considered important to sustain functional recovery after an SCI [9]. In contrast, typical movement disorders after SCI, e.g., spastic movement disorder, are due to the defective utilization of afferent input in combination with secondary compensatory mechanisms [27]. It has been shown that neuronal networks underlying the generation of locomotor patterns of cats [28] and humans [29,30] have an impressively high level of flexibility after SCI. Rehabilitative interventions after SCI should therefore focus on exploiting the plasticity of neuronal circuits, i.e., at supraspinal and/or spinal level, rather than focusing on improving isolated clinical signs, such as muscle tone or reflex excitability.

The plasticity of spinal neuronal circuits is task-specific and use-dependent as shown in several earlier experiments in cats with complete SCI. For example, after several months of daily step training, spinal cats regained full weight-bearing locomotion on a treadmill [31,32]. If a spinal cat is intensively trained to stand it develops the ability to support its body weight for up to an hour, but stepping ability on the treadmill remains poor [33]. These findings suggest that spinal neuronal circuits learn the sensorimotor task that is specifically practiced and trained [34]. The repetitive activation of particular sensorimotor pathways by task-specific training can reinforce circuits and synapses used to successfully perform the practiced movement [35,36]. Therefore, the outcome of a neurorehabilitative approach strongly depends upon the type, the repetition and the quality of the trained motor function.

Neuronal plasticity is not always positive, anatomical and neurophysiological observations in animals suggest that after SCI severed axons degenerate and create free synaptic territories which could become re-occupied by sprouting of intraspinal fibres [37]. The new neuronal circuits may be aberrant and can lead to inappropriate movement patterns or pain in rats [38] and humans [39]. This can largely be prevented by a combination of locomotor training, electrical and pharmacological stimulations of the region of the spinal cord that is deprived of supraspinal input which leads to a weight-bearing locomotor capacity in spinal rats [38]. For human SCI, it will be important to make sure that future neurorehabilitatitive approaches direct the spontaneous and/or experimentally induced (e.g., stem cells or Nogo-A antibodies) neuronal plasticity towards functional synaptic connections that are associated with an improved locomotor performance.

The decline in supraspinal and peripheral input in humans after a severe SCI is suggested to be responsible for the development of a neuronal dysfunction below the level of lesion [40,41]. Characteristic changes in neuronal behavior occur up to approximately one year after SCI. At one year post-injury complete and severe but incomplete SCIs are characterized by an exhaustion of leg muscle activity during assisted locomotion which is associated with changes in polysynaptic spinal reflexes [42]. Neuronal dysfunction after SCI is assumed to be dependent more on the subjects’ loss of mobility, i.e., decreased appropriate afferent information to the CPG, than on the completeness of the injury [43]. Consequently, the better the stepping ability of SCI subjects, the less the neuronal dysfunction. The functional state of spinal locomotor circuitries is not fixed after an SCI and neuronal dysfunction can be improved by intensive locomotor training over one month, but only in incomplete SCI subjects [43].

### Locomotor capacity after spinal cord injury

#### Locomotor pattern generation: from animal to human

Cats with a complete spinal cord transection at thoracic segments gradually improve hindlimb locomotion on a treadmill following 2–3 weeks of daily locomotor training [31,33]. The spinal cat can relearn walking with alternating steps in the hindlimbs, body weight support and plantar foot placement. Under such circumstances the EMG activity of the hindlimbs was remarkably similar before and after the spinal cord transection. However, the EMG amplitudes of leg extensors were smaller, whilst the timing between flexor muscles, such as hip and knee joint flexors, was changed and lead to foot dragging early in the swing phase [44]. With ongoing training it has been shown that the body support can be decreased and this is associated with improved locomotor capacity until a complete support of body weight and well-coordinated hindlimb stepping movements are possible [45]. Experiments in cats as well as non-human primates with complete spinal cord lesions have shown that the isolated spinal cord has the capacity to produce stepping patterns [46].

There are several indications for the existence of spinal neuronal circuitries for locomotion generation in humans. For example, step-like leg movements are present at birth and can be initiated spontaneously or by peripheral stimuli [47]. It is likely that the EMG activity underlying this newborn stepping is produced at the spinal level, as corticospinal projections are not fully grown and myelinated in newborns. In addition, rhythmic: coordinated leg movements have been observed in motor complete SCI subjects, during sleep [48] and after spinal cord stimulation [49,50].

#### Effect of locomotor training after SCI

**Figure 1:**
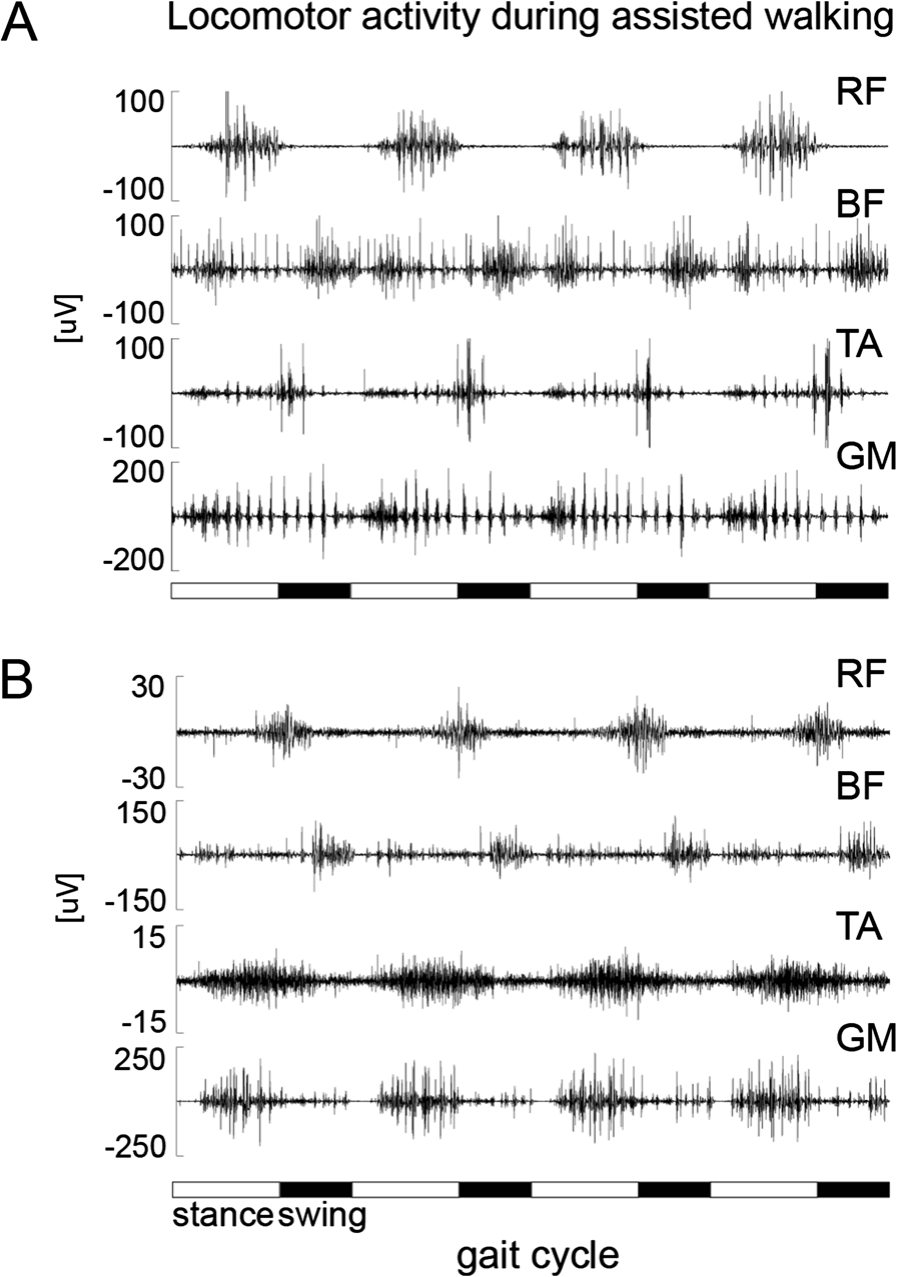
**Two examples of locomotor activity during assisted walking in the driven gait orthosis Lokomat in (A) an acute (3 months after SCI) and (B) a chronic (41 months after SCI) paraplegic subject.** Both SCI subjects suffered a motor complete spinal cord lesion and leg muscle activity was assessed in rectus femoris (RF), biceps femoris (BF), tibialis anterior (TA) and gastrocnemius medialis (GM) (modified from [42]).

In motor complete and incomplete SCI subjects a coordinated leg muscle activation pattern in both legs can be induced following partial unloading standing on a moving treadmill (Figure 1) [4,51]. In complete SCI subjects with no voluntary motor control below the level of lesion leg movements have to be assisted manually or by a robotic device during the whole training period. These subjects cannot relearn the ability to perform unsupported stepping movements on solid ground, but following such training they experience positive effects on cardiovascular and musculo-skeletal systems, such as a reduction of muscle spasms.

In the early phase of rehabilitation after a severe incomplete SCI, the main limitations for overground ambulation are usually reduced coordination, leg paresis, and impaired balance. Therefore, at the beginning of the training program locomotor training has to be assisted either by physiotherapists or a robotic device (e.g. Lokomat). During assisted locomotion in incomplete SCI subjects the induced leg muscle EMG activity is modulated over the step cycles in a similar way to as observed in healthy subjects. However, the amplitude of leg muscle activity is considerably smaller and corresponds to the degree of paresis probably due to a diminished noradrenergic input to spinal locomotor circuitries [45]. Providing full body unloading during robotic assisted walking in complete paraplegic subjects does not lead to significant leg muscle activation [52], i.e., ground contact is essential for leg muscle activation. During the course of an assisted locomotor training program, the EMG amplitudes of gastrocnemius activity increase during the stance phase, while inappropriate tibialis anterior activation decreases [53]. This leads to an improvement in weight bearing function during stance phase and thus allows a decrease of body unloading during assisted treadmill locomotion. The successive reloading of the SCI subjects might be an important stimulus for extensor load receptors which are essential for leg extensor activation during locomotion in cats [54] and humans [55,56].

The general rehabilitation strategy to regain locomotor ability after SCI or stroke is based on the principles of motor learning, such as task-specificity, task-variability, feedback information and the intensity of training. So far training paradigms for robotic assisted treadmill training in human SCI rely on suggestions by experts and single observational studies. However, no study has so far determined the essential components required for a locomotor training setup in individuals with SCI or in animal models of SCI. Several questions remain to be answered, for example, how early should the training therapy start, how intensive should it be, e.g., 30 min or more than 1 hour and how many times a week? It is not yet clear whether a longer duration of training results in an improved outcome or if certain endpoints, in terms of walking capacity, can be achieved within shorter periods of time. This question has so far only been addressed in stroke subjects where a longer duration of early onset augmented locomotor training, i.e., 16 hours within the first 6 months after insult, was correlated with improved walking performance (positive dose–response relationship) [57]. Whether this relationship is also applicable to the SCI community will be investigated by a controlled randomized multicenter study [58]. In addition, it remains to be determined which training paradigms fit the best to which SCI subjects and how this training could be adapted in difficulty, e.g., by the amount of body weight support, guidance force, treadmill speed. In stroke subjects higher treadmill speeds lead to better locomotor recovery than lower treadmill speeds [59]. It is assumed that these training principles which were applied in stroke subjects are also reasonable for successful training in SCI subjects. In general, locomotor training should always be challenging for patients with only minimal support provided by therapists or robotic devices.

#### Appropriate sensory cues

After an incomplete SCI, spared corticospinal and/or propriospinal pathways can play an active role in the recovery of locomotion. However, under these circumstances the intrinsic capacity of spinal locomotor circuitries and the sensory feedback information still remains as the basis for generating a locomotor pattern. The spinal locomotor circuitries interact dynamically with specific afferent inputs from receptors located in muscles, joints, and skin, and this interaction shapes the locomotor output (for review see [9]). The sensory input most relevant for locomotion comes primarily from stretch- and load-sensitive mechanoreceptors located in the muscles and skin. Afferent information from hip flexors and ankle extensors is particularly important for the stance to swing transition phase in cats [60]. Furthermore, sensory input from the skin mechanoreceptors of the paw are involved in positioning the paws and, therefore, become increasingly important during complex locomotor tasks in which precise paw placement is required, e.g., ladder walking in the cat [61]. Skin receptors on the dorsal foot play a role during the swing phase of walking over obstacles in both cats [62] and humans [63] and cutaneous input from the plantar surface of the paw reinforces extensor activity in decerebrated cats walking on a treadmill [64]. Group II hip flexor afferents, group I ankle extensor afferents, and low-threshold cutaneous afferents from the cat paw are assumed to have direct access to spinal locomotor circuitries with the ability to reset or entrain fictive locomotion in adult decerebrated cats [65].

**Figure 2:**
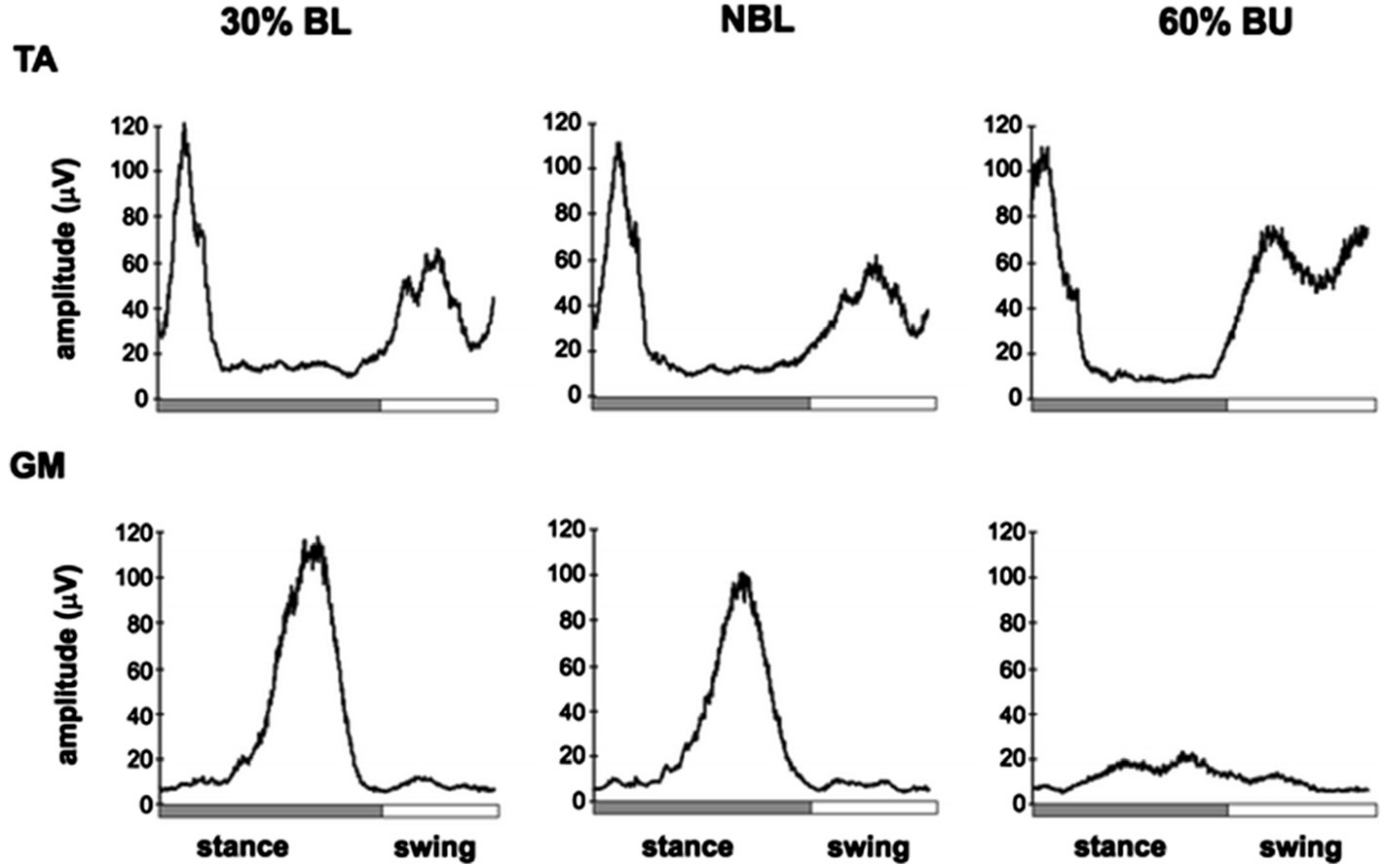
**Influence of body loading on leg muscle EMG activity.** Loading/unloading effects on the EMG activity of the left tibialis anterior (TA, ankle flexor) and gastrocnemius medialis (GM, ankle extensor). EMGs are the averages from seven healthy subjects. Three walking conditions are shown: normal body loading (NBL), 30% body loading (BL), and 60% body unloading (BU). In all subjects, there was a strong reduction of GM EMG activity during BU compared with NBL and 30% BL walking (modified from [67]).

Afferent inputs from load and hip joint receptors are essential for the activation of leg muscle activity during locomotion in paraplegic subjects in a corresponding way to in cats, [52,66]. Load information is provided for proprioceptive input from leg extensor muscles, namely Ib afferent signals from Golgi tendon organs, and probably also from mechanoreceptors in the foot sole (see Figure 2) [67]. This information is thought to be integrated into polysynaptic spinal reflex pathways that adapt the autonomous locomotor pattern to the actual ground condition and it is assumed that the Ib afferent input from leg extensors during the stance phase inhibits the flexor activity. This is functionally meaningful because loading of the stance limb has to decrease before swing can be initiated and leg extensor activity is reinforced during the stance phase by positive feedback [68]. In addition, the role of hip joint afferents is assumed to control phase transition and reinforce ongoing activity as it has been shown in decerebrated cats [65]. The observations that in motor complete paraplegic subjects assisted stepping movements within a driven gait orthosis and restricted movements of the hips (blocked knees) induces a patterned leg muscle EMG activity, highlights the significance of hip joint receptors in the generation of locomotor activity [52]. Such that assisted stepping movements restricted to imposed ankle joints were followed by no, or only focal reflex responses in the stretched muscles [52].

#### Modulation of spinal neuronal excitability

The importance of appropriate afferent information from peripheral receptors as a source of controlling locomotion became obvious from experiments, during the last decades, in completely transected animals. The inability to produce locomotor patterns after a severe SCI is, to a large extent, due to the depressed functional state of spinal locomotor circuitries [69]. It is believed that reduced sensory feedback after SCI has a negative impact on locomotor recovery. Therefore, there is a need to develop tools to artificially activate spinal cord pattern generators. Besides the essential sensory cues provided during locomotor training, i.e., from body loading and hip joint afferents, there has been much effort spent in developing additional strategies to increase the excitability of spinal neuronal circuitries in order to tune the physiological state of these circuitries to a level that leads to a facilitation of the locomotor patterns in humans. These approaches include continuous vibration of the quadriceps and hamstring muscle groups [70], continuous electrical stimulation of the peroneal or sural nerve [71], and magnetic stimulation of the spinal cord [72]. The latter approach of repetitive electromagnetic stimulation shows positive effects on spastic muscle tone in patients with diverse neurological diseases [73-75]. So far only one study investigated the effect of repetitive magnetic stimulation applied at the thoracolumbar vertebral level on spinal locomotor circuitries in healthy subjects [72]. The most favourable stimulation parameters to induce stepping-like activity in the legs placed in a gravity-neutral position were observed to be 3 Hz, 1.3 -1.82 tesla at the T11-12 vertebrae [72]. However, there is no proof that this kind of magnetic stimulation is able to evoke sufficient leg muscle activation to lead to load-bearing locomotion in SCI subjects.

In contrast to the low frequency (3 Hz) for thoracolumbar levels, higher frequencies (20–30 Hz) are necessary for peripheral nerve stimulation to decrease spastic muscle tone [74] and also for electrical epidural stimulation (~ 40 Hz) at the lumbar spinal cord in complete SCI subjects to induce stepping-like activity [49,76,77]. In addition to electrical or magnetic stimulation approaches, diverse pharmacological agents, such as serotonergic and noradrenergic agonists, can activate spinal locomotor circuitries in rats, cats and mice [38,78,79]. However, so far there is only limited evidence about whether pharmacological agents facilitate locomotor recovery following human SCI (for review see [80]). Recently, two new noninvasive methods, namely transcutaneous spinal direct current stimulation (tsDCS) [81-83] and paired spinal associative stimulations of H-reflexes and transcranial magnetic stimulation [84], have been applied to modulate spinal excitability and might be a promising tool for the modulation of sensorimotor pathways underlying functional movements.

TsDCS is a method derived from transcranial direct current stimulation and it influences neuronal excitability by anodal or cathodal polarization. Anodal stimulation typically increases neuronal firing rates in stimulated cortical areas, whereas cathodal stimulation evokes the opposite effect, i.e., inhibition. Depending on the duration and strength of polarization, these changes can persist for more than one hour after stimulation [85]. For the spinal approach the electrode positions are changed from the scalp to one electrode at T11 vertebral level and the other electrode on a shoulder region [81-83,86,88]. The polarization of the stimulation (anodal or cathodal) refers to the spinal electrode. TsDCS is applied for 15 min with a stimulation intensity of 2.5 mA. Depending on the electrode size (between 35 and 40 cm^2^) these stimulation parameters result in a total delivered charge of 0.056 and 0.064 C/cm^2^ respectively [81-83,86,87]. The dispersion and density of the current is, however, hard to predict due to the large volume of the conductor surrounding the target tissue. So far tsDCS has only been applied in healthy subjects as a tool to modulate trans-synaptic efficacy in monosynaptic [82,86] and polysynaptic reflex pathways [83], dorsal column function [81] or pain thresholds [87]. The effect on the excitability of spinal locomotor circuitries, especially in SCI subject, needs to be addressed in the future.

## Conclusion

Designing effective neurorehabilitation after SCI depends on having knowledge about the neuronal mechanisms involved in normal and pathological movement conditions, such as the interactions between central programs and afferent feedback as well as the quadrupedal coordination of human locomotion. Task-specific rehabilitation to improve locomotor function after SCI is well established [51,88]. In addition, there is growing evidence that the enhanced application of afferent input to the spinal cord, for example by electrical epidural stimulation of the spinal cord, can lead to a facilitation of standing and stepping-like activity during assisted leg movements in human SCI [77]. Consequently, it is argued that specific multi-pronged neurorehabilitative approaches will pave the way for more efficient therapeutic strategies to improve locomotor function in severely affected SCI subjects [89]. The aim of new neurorehabilitative approaches should be to optimize the use of task-specific sensory cues in order to facilitate locomotor pattern generation involving arm movements and the provision of approaches favouring the recruitment of both spinal circuitries and spared supraspinal connections during rehabilitation.

## Competing interests

The authors declare that they have no competing interests.

## Authors’ contributions

MH structured the content of the review and wrote the draft of the manuscript. VD made the revisions of the whole article. Both authors read and approved the final version of the manuscript.
